# Detection of *Acanthamoeba* Harboring *Campylobacter jejuni* Endosymbionts in Hospital Environments of Markazi Province, Iran

**DOI:** 10.1155/japr/6626888

**Published:** 2025-02-14

**Authors:** Alireza Mohammadi, Abdolhossein Dalimi, Fatemeh Ghaffarifar, Majid Pirestani, Majid Akbari

**Affiliations:** ^1^Department of Parasitology, Faculty of Medical Sciences, Tarbiat Modares University, Tehran, Iran; ^2^Department of Microbiology, Arak University of Medical Sciences, Arak, Iran

**Keywords:** *Acanthamoeba*, *Campylobacter jejuni*, endosymbionts, hospital samples

## Abstract

Most *Acanthamoeba*s contain endosymbionts such as viruses, yeasts, protists, and bacteria, some of which are potential human pathogens, including *Campylobacter jejuni* which often causes gastroenteritis and septicemia in humans. Amoebae have been shown to be resistant to chlorination and apparently protect ingested bacteria such as *C. jejuni* from free chlorine. Such resistance can have health implications, especially for drinking water treatment. The aim of this study is to identify *Acanthamoeba* in hospital samples in Markazi province, to determine the identity of *C. jejuni* endosymbiont in positive samples of *Acanthamoeba* in natural and laboratory conditions, and to determine the relationship between the two. The main aim of this study was to determine the identity of *C. jejuni* endosymbiont in *Acanthamoeba*-positive samples in natural and laboratory conditions. In this study, 134 samples including water, soil, and dust were collected from hospital environments. After molecular detection, the identity of the symbiotic *Campylobacter jejuni* in *Acanthamoeba* was determined by microscopic and PCR methods. Then, the ability of bacteria to infect the parasite was examined by cocultivation in vitro using real-time PCR. Finally, their relationship was examined based on statistical tests. The rate of contamination of hospital samples with *Acanthamoeba* was 44.7% on average. Out of 42 *Acanthamoeba* PCR-positive samples, seven isolates (16.67%) were found to be positive in terms of *C. jejuni* endosymbiont according to sampling location. The results showed that *Helicobacter* is able to penetrate and enter the *Acanthamoeba* parasite. In conclusion, our results showed that *C. jejuni* is able to contaminate *Acanthamoeba* in natural and laboratory conditions. The presence of pathogenic *Acanthamoeba* in various hospital environments and the hiding of *Helicobacter* as an endosymbiont inside it can pose a serious threat to the health of hospitalized patients.

## 1. Introduction

The concept of symbiosis among organisms encompasses various types of intercellular interactions such as mutualism, commensalism, and parasitism. Coexistence can manifest in two main forms: ectosymbionts, where the bacterium exists outside the cell or host organism, and endosymbionts, where the bacterium resides within another microorganism [1]. Identified bacterial endosymbionts naturally found in amoebae belong to diverse groups including Proteobacteria, Bacteroidetes, Flavobacterium, and Chlamydiae. Proteobacteria, the largest bacterial group, comprises subgroups like alpha, beta, gamma, delta, and epsilon [1]. Various endosymbionts, such as viruses, yeasts, protists, and bacteria, exist within *Acanthamoeba*s; some of these may be potential human pathogens, yet the precise nature and benefits of these symbiotic relationships remain largely unknown [2]. *Acanthamoeba* species can act as a reservoir for some pathogenic microorganisms and protect microorganisms from adverse environmental conditions [3]. Some bacteria can survive within amoeba cysts, evading chlorine and other disinfectants [4].

*Campylobacter jejuni*, classified within the Campylobacterales order of the Campylobacteraceae family and *Campylobacter* genus, possesses distinctive spiral shape and nonsheathed polar flagella [5]. These bacteria have a nonfermentative, nonoxidative metabolism and showcase significant genotypic and phenotypic diversity, inhabiting diverse ecological niches. Predominantly found in animals like cows and pigs, *C. jejuni* can cause infertility, abortion, and infections in poultry, particularly chickens, turkeys, and waterfowl [5].

*C. jejuni* stands out as a significant human pathogen among campylobacters, globally prevalent and frequently exceeding *Salmonella* or *Shigella* in diarrheal cases. It is primarily transmitted through contaminated food, especially undercooked poultry and water. In humans, it can cause gastroenteritis (the most common), toxic septicemia, and sometimes reactive arthritis and Guillain–Barré syndrome. *C. jejuni* has also been detected in people with septic arthritis, meningitis, and secondary proctocolitis. In numerous older or immunocompromised patients, it leads to bacteremia and is associated with immunoproliferative small intestinal disease (IPSID) [6, 7].

Moreover, *C. jejuni* infections are typically self-limiting, resolving within 3–7 days. Free-living amoebae, which feed on bacteria, serve as vital evolutionary cradles and genetic reservoirs for their internal microbes, facilitating genetic changes within the amoeba due to bacterial presence. These changes may include size augmentation to prevent predation, increased reproduction rates to avert species extinction, establishment of new ecological niches, and the emergence of antibiotic-resistant bacteria (ARB) [8].

Although the selective mechanisms in such symbiotic relationships are not fully understood, it appears that specificity is achieved through receptor recognition between the interacting organisms [9]. Knowledge surrounding the pathogenicity, drug resistance, and endosymbionts of amoebas, as well as their role in supporting bacterial pathogens, is limited [10]. Thus, studying the possible contamination of *Acanthamoeba* with *C. jejuni* endosymbionts in laboratory and hospital environments could enhance our understanding of amoeba–bacterial interactions and their complexities.

## 2. Materials and Methods

### 2.1. Sampling

A total of 134 samples including 112 (83.5%) dust samples, 11 (8.2%) soil samples, and 11 (8.2%) tap water samples were collected from hospital environments in Markazi Province, comprising seven hospitals in Arak City, one hospital in Farahan City, and another in Komijan City (Iran). These samples were gathered from various clinical units such as integrated comprehensive care (ICC), ICU, chemotherapy departments, oncology, heart and lung department, neurology, and ophthalmology department. Dust samples were obtained using sterile swabs from different surfaces like medical equipment, ventilation systems, windows, floors, sinks, and patient room surfaces. Soil and water samples were collected from the hospital yard and stored in sterile glass containers. The samples were then transported to the laboratory and immediately subjected to microscopic examination. Positive or suspected cases of *Acanthamoeba* were cultured.

### 2.2. *Acanthamoeba* Cultivation

A nonnutritive agar (NNA) culture medium dissolved in phosphate-buffered saline (PBS) with a 24-h culture of inactive *Escherichia coli* bacteria (xenic culture) was utilized to grow *Acanthamoeba* [11, 12].

### 2.3. Axenic Culture of *Acanthamoeba*

To observe the ability of *C. jejuni* bacteria to penetrate into *Acanthamoeba* in laboratory conditions, the parasite must be without the target bacteria and in the form of a trophozoite, which was obtained in this way through axenic [13, 14]. *Acanthamoeba* with negative PCR results of the above bacteria was cultured and purified by subculturing and migration method in *E. coli*–coated NNA medium, and antibiotic/antifungal [15] was used for toxin and in case of contamination with 3% HCl was washed three times and cultured in NNA [16]. A portion of the cysts was added to the NNA culture plate with 1 mL of inactivated *E. coli* bacterial suspension. After 2–3 days, the cysts were transformed into trophozoites and an amount of 200–500-*μ*L peptone yeast extract glucose broth (PYG) (Sigma–Aldrich, United States) [17] and the antibiotic gentamicin (100 *μ*g/mL) (Zahravi Pharmaceutical, Iran) and antifungal fluconazole (64 *μ*g/mL) (Zahravi Pharmaceutical, Iran) were used to remove the contamination, and after 2–3 days, the cysts turned to trophozoites and some of the axenic liquid medium (PYG) was added to the culture. By repeatedly changing the culture medium every 24 h and providing food, the trophozoite reached the logarithmic growth phase. Then, add about 3–5 mL of Page's saline to the petri dish and put it in the freezer for a few minutes and with a small tap and aspiration, the parasite was separated from the medium and slowly collected from the culture medium; then, it was centrifuged (1000–3000 rpm, for 10 min) and the supernatant was discarded and a suspension was prepared again with Page's saline [18].

### 2.4. Cultivation of Bacteria

Positive and standard strains of *C. jejuni* bacteria (ATCC 29428) were obtained from Pasteur Institute and transported to the laboratory in an anaerobic jar. Conventional blood agar (Columbia Agar II) culture medium was used for the propagation of *C. jejuni*, which is cultured in microaerophilic conditions (85% N_2_, 10% CO_2_, and 5% O_2_) for 24–48 h [7, 19].

First, the number of 3–5 isolated bacterial colonies on the culture medium was dissolved in 3–5 mL of PBS and its turbidity was adjusted with the concentration of half McFarland colony-forming unit (cfu per milliliter) (1 × 10^7^–10^8^). So that the standard turbidity density was determined by measuring absorption in a spectrophotometer with an optical path length of 1 cm. The half McFarland standard has an absorbance of 0.1–0.8 at the wavelength of 625 nm. *Acanthamoeba* to bacteria ratio multiplicity of infection (MOI) = 1 : 10 was prepared with PAS and used in the amount of 100–200 mL in coculture [17, 18].

### 2.5. Proximity of Bacteria and Parasites and Extraction of DNA From Coculture

The coculture stage was carried out according to the standard method [17, 18]. If the bacteria were not removed from the parasite body (checked by warm staining), the acid wash method (3% hydrochloric acid) was used [14]. To investigate the bacteria inside the *Acanthamoeba*, the acid wash technique (3% HCl) was used overnight (about 18 h) to remove foreign bacteria [20]. Finally, the sample was kept at −20°C until DNA extraction. DNA was extracted from the coculture according to the protocol of the DNG-Plus kit purchased from CinnaClone (Iran), and after extraction, the DNA concentration was measured by a nanodrop device [21].

### 2.6. DNA Extraction

Parasite DNA extraction followed the protocol of the DNG-Plus kit (CinnaGen, Iran) and was stored at −20°C until PCR testing. The DNA from T4 *Acanthamoeba* sp. suspension served as a positive control, while sterile distilled water acted as a negative control [22]. Qualitative assessment was done through 1% gel electrophoresis and quantitative analysis using a nanodrop device [22]. The absorbance ratios of 260/280 equal to 2 are considered a cutoff.

### 2.7. PCR

For *Acanthamoeba* testing, the PCR reaction employed the diagnostic primers JPD1 and JPD2 [11]. Additionally, a pair of primers targeting the *hipO* gene was designed for *C. jejuni* detection, amplifying the 438-bp fragment. These primers include forward primer *hipO*-F (AGTTATTGGAAGGGGTGGTC) and reverse primer *hipO*-R (CCAAAATCCTCACTTGCCAT) [22] (Table 1).

Bacterial primers were made by Gene Fanavaran (Iran) with a stock of 100 pmol/*μ*L in Tris-EDTA (TE) buffer; then, dilution was done at a concentration of 10 pmol/*μ*L and stored at −20°C. Distilled water was used for negative control, and DNA of *Acanthamoeba* and *C. jejuni* was used as a positive sample.

The PCR reaction for each sample was performed as follows: with a volume of 15 *μ*L containing 7.5 *μ*L Master Mix RED, 5.5 *μ*L template DNA (positive control: 1 *μ*L), and 1 *μ*L primer pair and 1 *μ*L buffer water (5.5 *μ*L in the positive control and 6.5 *μ*L in the negative control) and with conditions: denaturation initial at 94°C/5 min, the second stage of 40 main cycles (denaturation at 94°C/30 s, annealing at 47°C/30 s, and expansion at 72°C/30 s), and the third stage of final expansion at one cycle at 72°C/10 min thermocycler machine (Bio-Rad, United States), and in the next step, to measure the accuracy of the PCR product (5 *μ*L), a 100–3000 bp ladder was used as a DNA ladder (SMOBIO, Taiwan) and a 1.5% agarose gel plus 3 *μ*L SafeStain (DNAbiotech, Iran) and the size of PCR bands were successfully observed and analyzed by a UV device. And the same method was used for *Acanthamoeba* [10].

### 2.8. Parasite Contamination Under Lab Conditions

*Acanthamoeba* contamination with *C. jejuni* was quantified using the real-time PCR (RT Pcr) technique. To validate the findings, five iterations of bacterial and parasite cultivation, coculturing, and RT Pcr analysis were carried out.

### 2.9. RT Pcr

For quantitative measurement of *Acanthamoeba* DNA, JDP1 and JDP2 primers were used, but for quantitative measurement of *C. jejuni* endosymbiont DNA, *cadF* genes were used (to detect the 182-bp fragment), which includes cadF-F (TAAAAGCGGTGGATTTGGAC) and cadF-R (GCAGACATTTTGCTTGTGGA) (Table 1). In this way, the dilutions of the standard curve were first prepared and the copy number of DNA molecules was calculated based on the initial dilution of DNA using the website http://scienceprimer.com/copy-number-caculator-for-realtime-pcr.

For the standard curve and positive control, the confirmed DNA of *Acanthamoeba* and bacteria, as well as sterile distilled water, was used as a substitute for DNA in the negative control. The reaction solution with a volume of 20 *μ*L contained 10 *μ*L 2X Master Mix (SYBR Green), 2 *μ*L template DNA and 2 *μ*L primer pairs, and 6 *μ*L buffer water (8 *μ*L negative control), which was a ready-to-use solution for real-time quantitative measurement. RT Pcr reaction in “Stratgen MX3000p” device (Gilenya Company, Switzerland) during the following steps: (1) initial denaturation (one cycle 95°C/10 min); (2) 40 main cycles (denaturation 95°C/15 s, annealing 58°C/30 s for *Acanthamoeba* and 57°C/30 s for bacteria, and expansion 72°C/30 s); (3) a melting curve cycle (denaturation 95°C/15 s, annealing 60°C/20 s, and expansion 95°C/15 s) was performed.

Data analysis and the accuracy of RT Pcr product were checked by plotting the proliferation curve and melting curve. The data and results of each experiment were initially presented in the form of a proliferation curve in the form of a sigmoid diagram, where the *Y* axis indicates the amount of fluorescent signal and the *X* axis indicates the number of the reaction cycle.

To determine the absolute quantity of the template and the RT Pcr product, a standard curve was drawn for each *Acanthamoeba* and *C. jejuni*. The *R*^2^ value, slope, and efficiency value of standard curve were calculated 0.999%, −3.20%, and 104.9%, respectively. First, dilution was done with four to seven standard solutions; then, the number of copy molecules of all dilutions was calculated, and the dilutions made based on the copy number were entered into the device, and the RT Pcr reaction was performed for all dilutions prepared independently with a dedicated temperature program and with a volume of 20 *μ*m, and based on the amount of fluorescence in each tube, it is obtained by the proliferation chart device, and the Ct of the samples corresponding to each dilution was obtained from the proliferation curve. Based on Ct on the *Y* axis and log number of copies of the primary template based on serial dilutions, the standard curve was drawn on the *X* axis.

### 2.10. Statistical Analysis

The data was statistically analyzed using Excel and SPSS software (version 26). The relationship between *Acanthamoeba* infection and *C. jejuni* was assessed through two-way analysis of variance (ANOVA) and Pearson's correlation coefficient test, as well as simple linear regression. A significance level of *p* < 0.05 was deemed statistically significant.

## 3. Results

### 3.1. Microscopic Examination

*Acanthamoeba* were detected in 94 samples out of 134 (70.15%) by microscopic examination. The contamination rates were calculated as 76.2%, 83.3%, 42.1%, 90%, 77.8%, 45.4%, 88.9%, 80%, and 66.6% in Amirkabir, Qods, Khansari, Taleghani, Tamin Ejtemaei, Valiasr, Amiralmomenin, Farahan, and Komijan hospitals, respectively. Based on the chi-square statistical analysis (with a 95% confidence interval), a significant difference (*p* value = 0.010) was observed among the contamination rates in different hospitals.

### 3.2. *Acanthamoeba* Molecular Identification

PCR reactions were conducted on 94 positive microscopic hospital samples, revealing that 42 (44.7%) were positive with PCR. The most common genotype found in hospitals was *Acanthamoeba* T4 (11).

### 3.3. Molecular Results of *Acanthamoeba* Isolates in Relation to *C. jejuni* Contamination

Among the 42 hospital *Acanthamoeba* PCR-positive samples, seven isolates (16.67%) tested positive for *C. jejuni* endosymbiont. The highest positivity rate of 50% was observed in Khansari Hospital. No contamination was detected in Farahan, Komijan, Amirkabir, Valiasr, Qods, and Tamin Ejtemaei Arak hospitals. Statistical analysis indicates that the presence of *C. jejuni* in *Acanthamoeba*-positive samples did not vary significantly (*p* = 0.08), based on the sampling location. Detailed results are provided in Table 2.

Out of 42 positive *Acanthamoeba* PCR samples, seven isolates (16.67%) were found positive for *C. jejuni*, while 35 isolates (83.33%) were negative. Frequency of *C. jejuni* symbiosis with *Acanthamoeba* in different hospitals is shown in Table 2.

Soil samples showed no positive results, whereas the highest contamination rate (33.33%) was found in dust samples from general hospital departments. Statistical tests indicates that the presence of *C. jejuni* in positive *Acanthamoeba* samples did not show significant differences based on type and sampling unit (Table 3).

### 3.4. *Acanthamoeba* and *C. jejuni* Genotyping Results

As depicted in Table 4, *C. jejuni* was more prevalent in *Protacanthamoeba bohemica* compared to other *Acanthamoeba* genotypes. T2 had the highest occurrence, while T5 did not exhibit the mentioned endosymbiont. According to Fisher's test (*p* = 0.09), there was no significant association between genotype and the *C. jejuni* endosymbiont.

### 3.5. Results of Coculture in Lab Conditions

#### 3.5.1. RT Pcr Findings in Coculture

**Figure 1:**
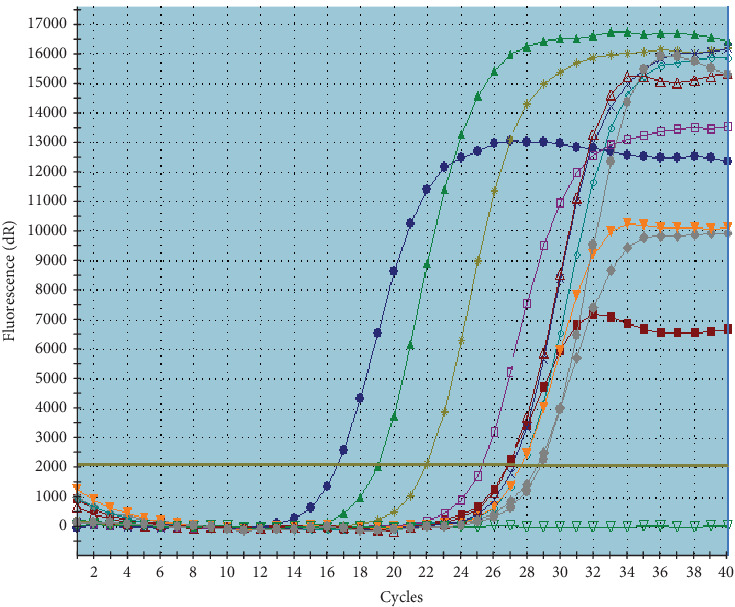
Amplification curve of *C. jejuni* in RT Pcr.

**Figure 2:**
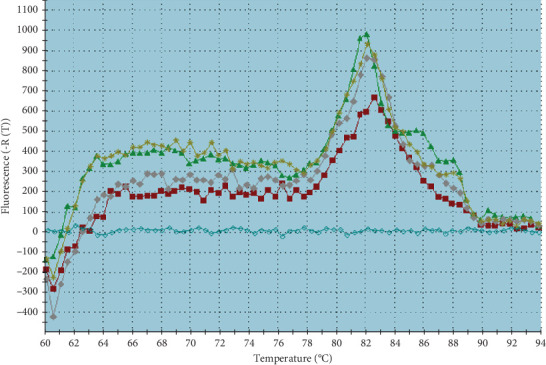
Melting curve of *C. jejuni* amplification in RT Pcr.

**Figure 3:**
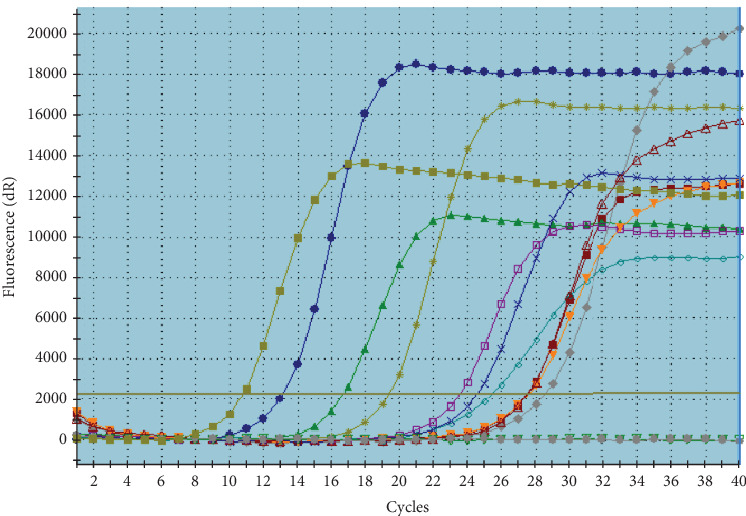
Amplification curve of *Acanthamoeba* in RT Pcr.

In laboratory settings, the penetration of *C. jejuni* into *Acanthamoeba* was examined using RT Pcr, with five technical replicates conducted. The accuracy of RT Pcr product was checked by plotting the amplification and melting curves for both *C. jejuni* and *Acanthamoeba* (Figures 1, 2, and 3), and the outcomes are detailed in Table 5. The molecular analysis of *C. jejuni*'s endosymbiont characteristics in lab conditions, as per Table 4, demonstrated the bacterium's capability to infiltrate and inhabit the *Acanthamoeba* parasite. The average penetration quantity of *C. jejuni* across five technical replicates was 3.18 × 10^6^, with a molecular ratio of *C. jejuni* to *Acanthamoeba* of 0.167.

### 3.6. The Correlation Between *Acanthamoeba* and *C. jejuni* Assessment

The correlation between *Acanthamoeba* and *C. jejuni* was assessed using the correlation test, while their linear relationship was examined with the regression test. The results in Table 6 indicate the following: Pearson's correlation coefficient (0.946) and *p* = 0.015 for *Acanthamoeba* and *C. jejuni* endosymbiont demonstrate a strong and significant correlation between these two microorganisms. Moreover, the ANOVA(*F*) or regression test with *p* = 0.015 suggests a noteworthy linear relationship between the parasite count and bacteria. The adjusted coefficient of determination (adjusted *R* square) reveals that the correlation between *Acanthamoeba* and bacteria stands at 86%.

## 4. Discussion

Most free-living amoebas are nonpathogenic; some, like *Acanthamoeba*, are implicated in both opportunistic and nonopportunistic infections of the human eyes, skin, and central nervous system. *Acanthamoeba* can act as a reservoir for some pathogenic bacteria and provide shelter and protection for the bacteria against adverse conditions [3]. These amoebae show resilience to chlorine and potentially protect against ingested bacteria such as *S. enteric*, *Yersinia enterocolitica*, *Shigella sonnei*, and *C. jejuni* [3]. The Environmental Protection Agency (EPA) lists 539 bacterial species. A thorough literature review indicates that 102 species (18.9%) can remain viable when interacting with various amoeba species. Among these, 40 species (39.2%) were isolated through amoeba cocultures but did not exhibit complete intracellular survival. It was also noted that 30 species (29.4%) survived within one or more amoeba species, while 32 species (31.4%) not only survived but thrived within one or more amoeba species. *C. jejuni* is among these bacteria, previously reported to replicate and persist intracellularly in free-living amoeba [21]. Some studies show that despite the need for microaerophilic conditions for cultivation in artificial media, *Campylobacter* spp. can survive and grow in the amoebae. This suggests that *Acanthamoeba* species can provide a suitable ecological environment for a variety of bacterial species [21].

The survival of *Campylobacter* spp., particularly *C. jejuni*, in environmental settings such as soil, dust, and water varies based on several factors, including temperature, moisture, and the presence of organic matter. Different *Campylobacter* isolates have been shown to vary in their ability to survive in water [23]. Survival in water was temperature dependent, with *Campylobacter* generally surviving much better at low temperatures (10°C–16°C) than at room temperature. Similarly, different strains of *C. jejuni* from different origins have shown an origin-dependent ability to survive in sterilized drinking water [24]. *C. jejuni* strains can also survive for long periods in well water [25]. Hutchison et al. [26] demonstrated recovery of *C. jejuni* from contaminated soil after a period of 34 days in an outdoor study in the United Kingdom.

*Acanthamoeba* is ubiquitous in various environments and can be found in many natural and artificial settings such as water, soil, and biofilms in various settings like hospitals and clinics. Molecular investigations in our study revealed that *Acanthamoeba* samples from the hospital environment were infected with *C. jejuni* bacteria and its respective endosymbiont. Based on sample type, no cases were detected in soil samples. In terms of sampling units, the hospital's general wards (including admission, triage area, injection room, laboratory, drug store, hemodialysis, radiology, and hematology) exhibited the highest presence of the *C. jejuni* endosymbiont.

*C. jejuni* was shown to invade and colonize in the *Acanthamoeba* in vitro (coculture). RT Pcr quantification indicated a significant correlation between the quantities of *Acanthamoeba* and *C. jejuni* in the coculture. Our study's findings further confirm *C. jejuni*'s significant ability to survive and reproduce within *Acanthamoeba*. A study found that cocultivating *Campylobacter* with *Acanthamoeba castellani* aided its survival and growth, although the bacterium was unable to replicate inside the amoeba alone. The authors concluded that the presence of *A. castellani* stimulates the growth of this microaerobic bacterium by reducing dissolved oxygen levels in the environment [27]. Previous studies demonstrate that amoebae can create a conducive nutrient-rich and anaerobic environment for bacteria. While some studies focus on bacterial survival, others highlight their ability to multiply within *Acanthamoeba*, showing a lack of consensus in this area.

A laboratory study revealed that four different strains of *C. jejuni* could infect *Acanthamoeba polyphaga*. *Acanthamoeba* spp. are reported to be a transient host for *C. jejuni*, and survival of this bacterium in the amoebae enhances its invasion and subsequent survival in human epithelial cells and amoeba cells [28]. *C. jejuni* showed prolonged survival when cocultured with amoebae compared to monoculture, with bacteria remaining alive and motile within amoeba vacuoles, indicating that free amoebas may serve as a reservoir for *C. jejuni* in the environment [7]. These findings align somewhat with our own results.

Hojo et al. [29] suggested that *C. jejuni*'s survival in coculture depends on the reduced partial oxygen pressure created by the amoeba. Some ARB like *C. jejuni*, *Listeria monocytogenes*, and *Bacillus cereus* have been observed to survive extracellularly in coculture with amoebae [30].

Several laboratory studies have demonstrated that *Acanthamoeba* can facilitate the survival and growth of various human pathogenic bacteria [30]. Previous research indicates that *Campylobacter* tends to proliferate in the presence of *Acanthamoeba* in controlled laboratory settings. Despite conflicting findings on whether the interaction is intracellular or extracellular, particularly with *C. jejuni*, this may vary based on the bacterial strain and experimental techniques employed [31].

Our findings show that *C. jejuni* is present within *Acanthamoeba* in hospital settings, as evidenced by samples collected. Therefore, understanding the health risks associated with *Acanthamoeba* can significantly inform healthcare strategies and interventions through various means, such as patient awareness and education; training of healthcare workers; regular monitoring of hospital water, dust, and soil; sanitation of medical facilities and equipment; implementation of surveillance systems for early detection of *Acanthamoeba* infections; and development of case reporting protocols for *Acanthamoeba* infections, all of which will allow for better tracking of potential epidemiological trends and risk factors. By integrating this information into healthcare practices and policies, hospitals can increase patient safety, reduce the incidence of these infections, and improve overall health outcomes. However, more extensive research is needed, particularly focusing on the genetic features that influence its interaction and survival within the amoebae.

## 5. Conclusion

*Acanthamoeba* can act as a host and vector for various pathogenic microorganisms, including *Campylobacter*. This can lead to potential outbreaks, particularly in settings like hospitals where vulnerable populations may be exposed. In our laboratory experiments, it was observed that *C. jejuni* can contaminate, infiltrate, replicate, and endure within *Acanthamoeba*, thus validating their interplay even in natural settings. These outcomes bolster the theory that *Acanthamoeba* could serve as an environmental reservoir for *C. jejuni* bacteria. Consequently, vigilance is crucial concerning the presence of free-living amoebae in natural and healthcare settings; aside from the inherent risks posed by *Acanthamoeba*, their presence may enhance the bacterial durability, propagation, and survival in the environment, posing a potential health hazard.

## Figures and Tables

**Table 1:** Genes and primers for amplification of *Acanthamoeba* and *C. jejuni* in PCR and qPCR.

**Microorganism**	**Test**	**Target gen**	**Primer**	**Tm (°C)**	**Base no.**	**Oligo sequence 5**⁣′**→3**⁣′	**Size (bp)**
*Acanthamoeba*	PCR and qPCR	*18S rDNA* *ASA-S1*	JDP1 JDP2	56.7 57.1	22 24	GGCCCAGATCGTTTACCGTGAA TCTCACAAGCTGCTAGGGGAGTCA	450-500

*C. jejuni*	PCR	*hipO*	hipO-F hipO-R	51.8 49.7	20 20	AGTTATTGGAAGGGGTGGTC CCAAAATCCTCACTTGCCAT	438
	qPCR	*cadF*	cadF-F cadF-R		20 20	TAAAAGCGGTGGATTTGGAC GCAGACATTTTGCTTGTGGA	182

**Table 2:** Frequency of *C. jejuni* symbiosis with *Acanthamoeba* in different hospitals.

**Hospitals**	**Symbiosis between**		
	***Acanthamoeba***	***C. jejuni***	
	**Positive no. (%)**	**Positive no. (%)**	**Negative no. (%)**
Komijan	2 (3.33%)	0	2 (100%)
Farahan	1 (12.5%)	0	1 (100%)
Amirolmomenin Arak	10 (62.5%)	2 (20%)	8 (80%)
Taleghani Arak	7 (38.9%)	2 (28.66%)	5 (71.4%)
Amirkabir Arak	6 (37.5%)	0	6 (83.3%)
Khansari Arak	6 (75%)	3 (50%)	3 (50%)
Valieasr Arak	5 (50%)	0	5 (100%)
Qods Arak	2 (40%)	0	2 (100%)
Tamin Ejtemaei Arak	3 (42.8%)	0	3 (100%)
Total	42 (100%)	7 (16.67%)	35 (83.33%)

**Table 3:** Frequency of *C. jejuni* symbiosis with *Acanthamoeba* based on sampling type and unit.

**Samples**	**Symbiosis between**		
	***Acanthamoeba***	***C. jejuni***	
	**Positive no. (%)**	**Positive no. (%)**	**Negative no. (%)**
Dust from			
General wards^a^	15 (52.6%)	5 (33.33%)	10 (66.67%)
Specialized wards^b^	20 (45.4%)	2 (10%)	18 (90%)
Service departments^c^	5 (41.6%)	0	5 (100%)
Total	40 (48.2%)	7 (17.50%)	33 (82.50%)
Soil	2 (25%)	0	2 (100%)
Water	0	0	0
Total	42 (100%)	7 (16.67%)	35 (83.33%)

^a^General wards: admission, triage area, injection room, laboratory, drug store, hemodialysis, radiology, and hematology.

^b^Specialized wards: department of psychiatry, orthopedic, heart and lung, children, infants, midwifery, ICCU, CCU, ENT, surgery, ophthalmology, and mammography.

^c^Service departments: kitchen, laundry, CSR, equipment, and emergency wards.

**Table 4:** Frequency of *C. jejuni* symbiosis with different genotypes of *Acanthamoeba* and *Protacanthamoeba bohemica.*

**Free-living amoeba**	***C. jejuni* contamination**		**Total**
	**Positive no. (%)**	**Negative no. (%)**	
*Protacanthamoeba bohemica*	8 (57.14%)	6 (42.85%)	14 (46.7%)
T2 *Acanthamoeba*	2 (66.67%)	1 (33.33%)	3 (10%)
T11 *Acanthamoeba*	1 (50%)	1 (50%)	2 (6.7%)
T4 *Acanthamoeba*	1 (10%)	9 (90%)	10 (33.3%)
T5 *Acanthamoeba*	0 (0%)	1 (100%)	1 (3.3%)
Total	12 (40%)	18 (56.7%)	30 (100%)

**Table 5:** *Acanthamoeba* and *C. jejuni* DNA copy numbers in coculture (RT Pcr results).

**Test**	**DNA copy number**		
	***C. jejuni***	***Acanthamoeba* spp.**	**Ratio**
1	2.58 × 10^6^	1.58 × 10^7^	0.16
2	1.60 × 10^6^	7.25 × 10^6^	0.22
3	5.90 × 10^6^	8.23 × 10^7^	0.07
4	3.38 × 10^6^	1.47 × 10^7^	0.23
5	2.16 × 10^6^	1.53 × 10^7^	0.14
Mean	3.18 × 10^6^	2.70 × 10^7^	0.16
Positive control	3.42 × 10^5^	6.80 × 10^12^	

**Table 6:** Correlation and linear relationship between *Acanthamoeba* and endosymbionts.

**Correlations and regression**			
***Acanthamoeba* and endosymbiont**	**Value test**	**(Sig. 2-tailed)**	**Adjusted ** **R** ** square**
*Acanthamoeba* and *Campylobacter jejuni*			
Pearson correlation	0.946	0.015	0.860
ANOVA(*F*)/regression	25.593	0.015	

## Data Availability

The data used to support the findings of this study are available from the corresponding author upon reasonable request.
